# Hidden Treasure: Congenital Adhesions Necessitating an Alternative Approach to Laparoscopic Appendicectomy

**DOI:** 10.7759/cureus.35450

**Published:** 2023-02-25

**Authors:** Bichen Zhao, Peter Rogers, Helen Ballal

**Affiliations:** 1 General Surgery, Royal Perth Hospital, Perth, AUS

**Keywords:** intraabdominal adhesions, open and laparoscopic surgery, congenital adhesions, atypical appendicitis, general emergency surgery

## Abstract

Congenital adhesions are rare findings in adults. We present a case of appendicitis in a middle-aged male with extensive congenital adhesions of the terminal ileum to the right lateral abdominal wall. The small bowel mesentery completely obscured the inflamed appendix. Alternative techniques were required as a result of these intraoperative discoveries, and substantial adhesiolysis was carried out before a successful appendicectomy. Although the congenital adhesions described here are extremely uncommon, the authors suggest that practitioners should be aware of them because they could change typical clinical manifestations and surgical approaches.

## Introduction

Adhesions between the small bowel and other intra-abdominal structures usually form after abdominal inflammation such as post-surgery, radiation, or pelvic inflammatory disease [1]. Adhesions can lead to chronic abdominal pain, small bowel obstruction, and increased difficulty in surgical access [2].

Congenital intra-abdominal adhesions are a rare phenomenon usually observed in children. Most reported congenital adhesions are discovered during surgery for small bowel obstruction where a congenital band causes incarceration and strangulation of the small bowel as it passes through [3].

To the best of our knowledge, significant congenital adhesions of the small bowel itself to the abdominal wall have not been reported in the literature. This is an important entity for clinicians to be aware of, as it could alter the clinical presentation of common pathologies and increase the difficulty of an otherwise standard operation.

We present the case of a 40-year-old male who presented with acute appendicitis. During the laparoscopic appendicectomy, extensive congenital small bowel adhesions were encountered, which increased the difficulty of surgical access.

## Case presentation

The patient was a 40-year-old male who presented to the emergency department with three days of migratory right iliac fossa pain. It was constant in nature and worse on movement. He did not have any nausea or vomiting, no changes in bowel habits, and no subjective fevers. Apart from the pain, he stated that he felt well, without other systemic features of infection.

The patient’s only past medical history was diabetes and hyperlipidemia, which were both well-controlled on oral medications. He reported no past intra-abdominal surgery or trauma, and no past colonoscopies or gastroscopies. He did not smoke or drink alcohol.

On examination, his vital signs were within normal limits. His abdomen was mildly distended, and he was guarding in the right iliac fossa. He had equivocal rebound tenderness. He did not have Rovsing’s sign or psoas's sign present. He had mild percussion tenderness at the right iliac fossa.

A contrast-enhanced CT demonstrated a thickened and dilated appendix with associated fat stranding, consistent with appendicitis. At the time, no abnormal features of the small bowel were identified on imaging. Interestingly, the patient’s laboratory results showed very minimal inflammation, which was incongruent with the CT findings.

He had a normal white cell count and a C-reactive protein (CRP) of only 16. All other laboratory parameters are within normal limits.

A diagnosis of appendicitis was made, and the patient was started on intravenous broad-spectrum antibiotics. Arrangements were made to proceed to the operating theater for laparoscopic appendicectomy.

**Figure 1:**
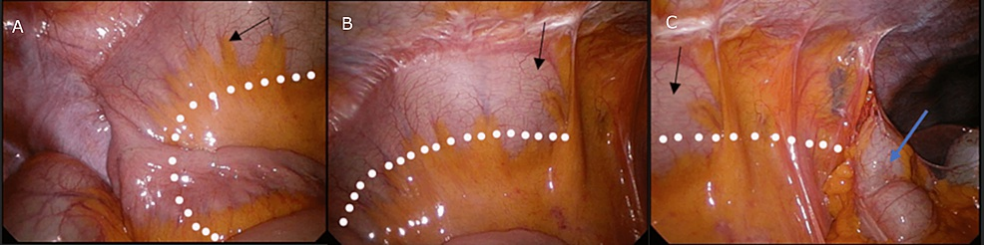
Intra-abdominal findings on entry Extensive adhesions of the terminal ileum (black arrow) to the right lateral abdominal wall. The transverse colon (blue arrow) can be found in the right upper quadrant. The obscured ascending colon is represented by the dotted line. Right lower quadrant (A), right lumbar (B), right upper quadrant (C)

Once pneumoperitoneum and visualization were established, it was noted that the ascending colon and the cecum were not visible. Upon further inspection, it was noted that a segment of the small bowel congenitally adhered to the right lateral abdominal wall (Figure 1).

**Figure 2:**
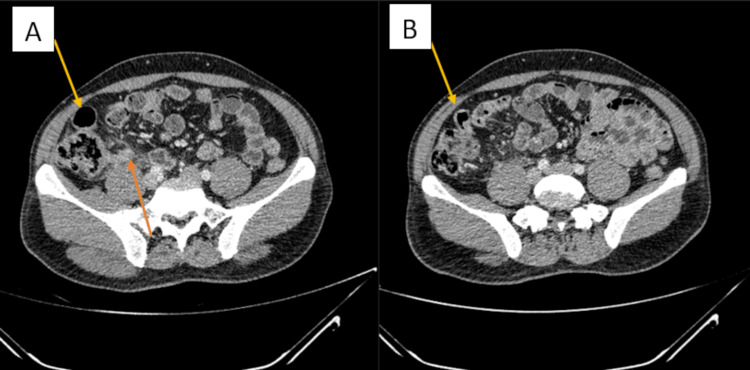
Pre-operative CT images show acute appendicitis; there were no findings that could indicate the congenital adhesions encountered on laparoscopy A: Terminal ileum (yellow arrow) is close to the abdominal wall but not shown to be adhered to it. Acute appendicitis is indicated by the orange arrow. B: Continuation of A, the adhered segment of the small bowel is the terminal ileum (yellow arrow) as it enters the cecum.

**Figure 3:**
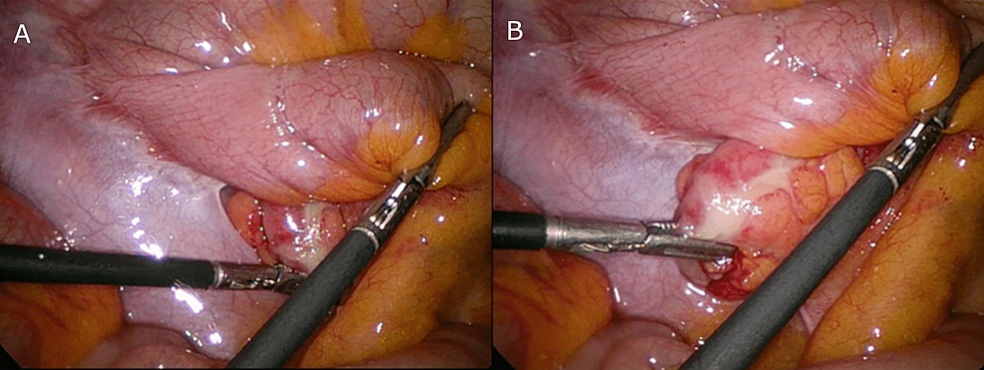
The obscured appendix can be found underneath the adhered terminal ileum A: Minimal retraction; B: Significant retraction

**Figure 4:**
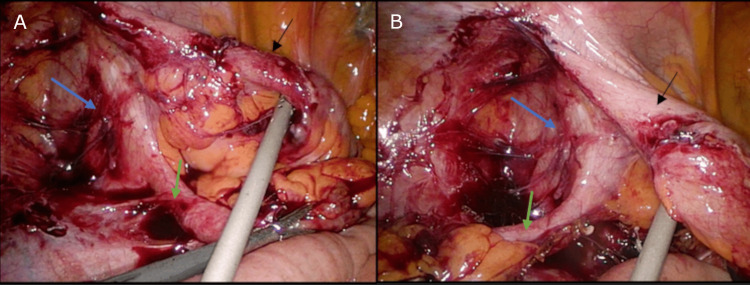
View of the full appendix post adhesiolysis of the terminal ileum Terminal ileum (black arrow), appendix (green arrow), cecum (blue arrow) A: Appendix retracted to the left iliac fossa; B: Appendix retracted to the right iliac fossa

The appendix, cecum, and ascending colon were completely obscured under the mesentery of this small bowel. It was clear that the infection and inflammation were well contained under the small bowel mesentery. Comparing intra-operative images with the preoperative CT scan, it can be determined that this segment of the small bowel is the terminal ileum. As the appendix and cecum were tucked far under the terminal ileum mesentery (Figure 2), the decision was made to perform an adhesiolysis to improve exposure. A standard appendicectomy was then able to be performed (Figure 3). Figure 4 shows the full appendix post adhesiolysis of the terminal ileum.

The patient was discharged from the hospital two days after the operation when bowel function returned. The subsequent 30-day follow-up revealed no complications and he was discharged from the clinic.

## Discussion

Acute appendicitis is one of the most common causes of acute abdomen and is one of the most common surgically treated pathologies [4]. Intra-abdominal adhesions can lead to difficult surgical access, causing an increase in surgical risk and complications. Mavros et al. found that the presence of concomitant adhesions leads to increased operative time, morbidity, and increased risk of inadvertent enterotomies [5]. In our case, the unusual finding of congenital adhesion led to increased surgical difficulty during the operation. To date, most congenital adhesions described are in the context of a band adhesion causing obstruction; to our knowledge, adhesions described in this case have not been previously reported.

The congenital adhesions encountered could also explain the unusual preoperative biochemistry. It was noted the patient only had a small increase in inflammatory markers; this was despite significant stranding on CT and a three-day history. The authors believe that these atypical findings were a result of the small bowel mesentery containing the infection. Interestingly, there have been other cases reported where an inflamed appendix was found in unusual spaces in the abdomen, leading to unremarkable changes in inflammatory markers. Probert et al. describe a case of appendicitis in an Amyand hernia in which the patient had down-trending inflammatory markers without treatment [6]. Sigley et al. also describe a case of appendicitis in an umbilical hernia where the inflammatory markers were also normal [7].

Our case also highlights a distinct advantage of a laparoscopic appendicectomy compared to open surgery. Traditionally, both open and laparoscopic appendicectomies are considered safe and effective in treating appendicitis [8]. Both methods have distinct advantages and disadvantages [9,10]. The authors believe that a traditional right iliac fossa incision would have been insufficient in this case given the appendix was tightly tucked under the terminal ilium mesentery. If access was obtained with a right iliac fossa (RIF) incision, it is likely the operation would be converted to a laparotomy to safely expose the appropriate structures. Laparoscopic surgery allowed full visualization of the abdominal cavity and safe adhesiolysis.

## Conclusions

Acute appendicitis is one of the most common emergency general surgery presentations to the emergency department. Patients mostly present with raised inflammatory markers and localized peritonism, especially if they have been symptomatic for more than 24 hours. In our case, the patient did not have these significant findings despite 72 hours of pain. This case highlights the importance of clinician awareness of the varied presentations in abdominal pathologies and the possibilities of congenital adhesions causing these variations. Although the congenital adhesions described in this case are extremely rare, clinicians should not be falsely reassured by benign inflammatory markers and incongruent examinations.
